# Beyond Glycemic Control: Concurrent GLP-1 Receptor Agonist Use Is Associated with Reduced Urinary Adverse Events Following OnabotulinumtoxinA Treatment in Non-Diabetic Adults with Overactive Bladder

**DOI:** 10.3390/toxins17110542

**Published:** 2025-11-01

**Authors:** Muhammed A. M. Hammad, Sophia G. Quesada, Aimee L. Belczyk, Gamal M. Ghoniem

**Affiliations:** Department of Urology, University of California Irvine, Orange, CA 92868, USA; mahammad@hs.uci.edu (M.A.M.H.); sgquesad@hs.uci.edu (S.G.Q.); abelczyk@uci.edu (A.L.B.)

**Keywords:** overactive bladder (OAB), GLP-1 receptor agonist, onabotulinumtoxin A, diabetes, semaglutide, weight loss, urinary retention

## Abstract

Semaglutide, a GLP-1 (glucagon-like peptide-1) receptor agonist, is widely prescribed for weight loss in non-diabetic populations. Given the link between obesity and overactive bladder (OAB), we explored whether GLP-1 use would improve adverse urinary events beyond its weight loss benefit for non-diabetic adults undergoing onabotulinumtoxin A (BTX-A) treatment for OAB. Using the TriNetX database, we conducted a retrospective cohort study of non-diabetic OAB patients treated with BTX-A alone or with concurrent GLP-1 therapy. Propensity score matching (1:1) was adjusted for age, race, ethnicity, hypertension, and BMI/obesity. After matching, 992 patients were included in each group. GLP-1 use was associated with a lower incidence of urinary retention (8.6% vs. 4.9%, risk difference 3.66%, *p* = 0.0044) and urinary tract infection (13.3% vs. 8.8%, risk difference 4.54%, *p* = 0.00224), with corresponding improved one-year retention-free and UTI-free survival on Kaplan–Meier (KM) analysis. Antispasmodic initiation rates were similar (11.8% vs. 10.3%, risk difference 1.55%, *p* = 0.6921), and KM analysis showed no significant difference. These findings suggest that GLP-1 receptor agonist use may improve select urinary adverse events in non-diabetic adults undergoing BTX-A treatment for OAB and support further investigation into its potential adjunctive role in OAB management with longer follow-up.

## 1. Introduction

Overactive bladder syndrome (OAB) is defined as urinary urgency, often accompanied by urinary frequency and nocturia, with or without incontinence. This urinary urgency can significantly interrupt a patient’s daily activities and sleep, profoundly diminishing their quality of life. The global prevalence of OAB is reported to be nearly 20%, with higher rates among female, elderly, overweight and obese populations [1]. With rates this high, several treatment strategies are offered to help patients manage their symptoms. Treatments vary in intensity, ranging from conservative measures to more advanced options, including pelvic floor exercises to strengthen the pelvic floor muscles that suppress urgency, and lifestyle therapies to mitigate the worsening effects of smoking and weight [2]. Pharmacological options, such as beta-3 adrenergic agonist drugs and anti-muscarinic agents, are common adjuncts to these primary treatments. Minimally invasive procedures, like percutaneous tibial nerve stimulation or sacral neuromodulation, may substitute or replace medical therapy if not effective [3]. When all else fails and symptoms persist, surgical interventions like augmentation cystoplasty and urinary diversion may provide relief to these refractory patients, albeit with variable results and significant potential for complications. Among minimally invasive therapies, intradetrusor onabotulinumtoxinA (BTX-A) injection has become an established and guideline-endorsed option for refractory OAB by recent guidelines from both the American Urological Association (AUA) and the European Association of Urology (EAU) [4,5,6].

Within 15 years, BTX-A has emerged as an option for refractory OAB by inhibiting neurotransmitter release and leading to reduced bladder overactivity [4]. Though effective, BTX-A is associated with an increased risk of elevated post-void residual volume and urinary tract infection (UTI) [7,8,9]. When compared against neuromodulation treatments like sacral nerve neuromodulation and percutaneous tibial nerve stimulation in a meta-analysis, BTX-A was still associated with more complications of urinary retention and UTI [10]. Thus, these adverse effects can necessitate intermittent catheterization for patients and may diminish the overall benefits of the treatment [11]. Especially for those populations who have high incidences of UTIs like the elderly—30% of women over the age of 85 report a UTI in the last 12 months—and obese patients, BTX-A injection benefits may be reduced [12,13,14].

The association between being overweight and OAB severity is well-documented [15,16,17]. Though the pathophysiology is not yet fully understood, many proposed mechanisms have been investigated. One mechanism describes how larger waist circumference and BMI may cause excess abdominal pressure, elevating bladder pressure and urethral mobility, further exacerbating OAB [18]. Another proposed mechanism ascribes the weakened pelvic floor brought about by obesity as the culprit [19]. Additionally, weight loss has been shown to reduce the severity of OAB symptoms, supporting obesity’s role as a modifiable risk factor in its pathogenesis [20].

Glucagon-like peptide-1 receptor agonists (e.g., semaglutide and liraglutide) (GLP-1 RA) were first approved by the Food and Drug Administration (FDA) in 2005 for the treatment of Type 2 Diabetes but have only recently revolutionized the market as a weight loss drug for the non-diabetic patient. Importantly, GLP-1 RA has been shown to significantly reduce weight with an acceptable safety for obese or overweight patients without diabetes [20]. By mimicking the effects of endogenous GLP-1, these drugs stimulate insulin release from the pancreas, regulate appetite and satiety in the brain, and slow down gastric emptying [21].

With this in mind, we wanted to explore if the benefits of GLP-1 RA reach beyond simply weight loss. Specifically, we hypothesized that GLP-1 receptor agonist use could modulate urinary adverse events following BTX-A, independent of glycemic control. In diabetic patients, these drugs have been shown to reduce albuminuria in chronic kidney disease, reduce all-cause mortality in cardiovascular disease, reduce blood pressure and dyslipidemia, and decrease metabolic complications in polycystic ovarian syndrome (PCOS) [22]. In non-diabetic patients, there is less direct though intriguing support for its renoprotective, cardiovascular, and fertility effects [22,23,24,25]. Other research highlights a potential anti-inflammatory effect, reducing circulating inflammatory cytokines like tumor necrosis factor α (TNF-α) and interleukin-6 (IL-6), and highly sensitive C-reactive protein, in both mice and human models [26,27,28,29]. As inflammation, autonomic tone, and detrusor overactivity all contribute to OAB, we speculate that GLP-1 RA could additionally influence bladder function.

So far, no studies have evaluated urinary outcomes of GLP-1 RA in non-diabetic OAB patients on BTX-A. This retrospective cohort study aims to explore the potential impact of GLP-1 receptor agonist use on urinary outcomes in non-diabetic patients with refractory OAB undergoing onabotulinumtoxinA treatment, addressing a critical gap in the current literature. Using the TriNetX database, we conduct a large-scale analysis highlighting any discrepancies in outcomes between GLP-1 RA users and control cohorts.

We hypothesized that GLP-1 RA is associated with improved urinary adverse effects in non-diabetic patients with OAB receiving BTX-A beyond weight loss benefits. Primary outcomes evaluated the incidence of urinary tract infection (UTI), urinary retention, and urinary antispasmodic use following BTX-A treatment.

## 2. Results

A total of 19,907 OAB patients who received BTX-A alone and 992 OAB patients who received both BTX-A and a GLP-1 receptor agonist (GLP-1 RA) were identified before matching. After 1:1 propensity score matching, 992 matched pairs were included for analysis.

The median follow-up duration was 365 days for the BTX-A-only cohort (IQR: 77.5 days) and 365 days for the combination cohort receiving BTX-A plus a GLP-1 receptor agonist (IQR: 163.5 days). The mean age was nearly identical (59.3 vs. 59.4 years; *p* = 0.8073). After matching, female patients comprised 913 (92.0%) of the BTX-A-only cohort and 904 (91.1%) of the BTX-A + GLP-1 RA cohort (*p* = 0.4668), while male patients accounted for 59 (5.9%) and 63 (6.4%), respectively (*p* = 0.7085), indicating comparable sex distributions between groups. The prevalence of cystitis was higher in the GLP-1 group (34.2% vs. 30.1%; *p* = 0.0545), though this did not reach statistical significance (Table 1).

Medication utilization after matching revealed higher use of antimicrobials in the GLP-1 group (93.5% vs. 98.4%; *p* < 0.0001), as well as increased use of antiemetics (75.1% vs. 83.9%; *p* < 0.0001) and laxatives (68.5% vs. 74.6%; *p* = 0.0028).

### 2.1. Urinary Retention

For urinary retention outcomes, 212 patients in the BTX-A-only cohort and 241 patients in the BTX-A + GLP-1 RA group were excluded due to a documented history of urinary retention prior to receiving BTX-A or BTX-A + GLP-1 RA in each group, respectively. Among the remaining patients, retention occurred in 67 of 780 patients (8.59%) in the BTX-A-only cohort, compared to 37 of 751 patients (4.93%) in the combination cohort. This translated to an absolute risk difference of 3.66% (95% CI: 1.16–6.17%; *p* = 0.0044).

**Figure 1:**
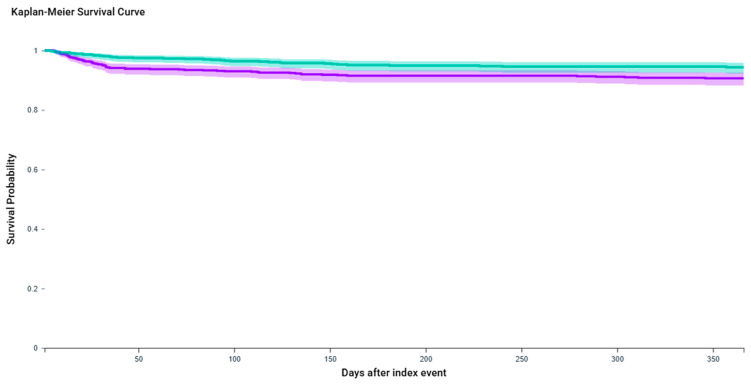
Kaplan–Meier curves for urinary retention-free survival over one year following BTX-A injection, with or without concurrent GLP-1 receptor agonist therapy. The purple line represents the *OAB + BTX-A injection* cohort, while the teal line represents the *OAB + BTX-A + GLP-1 agonist/Semaglutide* cohort. Patients treated with combination therapy (OAB + BTX-A + GLP-1 agonist) had significantly higher retention-free survival compared to those receiving BTX-A alone (log-rank *p* = 0.0064). At one year, the retention-free survival probability was 94.3% in the combination group versus 90.6% in the BTX-A-only group. The hazard ratio for urinary retention in the BTX-A-only group was 1.74 (95% CI: 1.16–2.59), indicating a 74% increased risk relative to combination therapy.

Kaplan–Meier (KM) analysis, excluding patients with pre-existing retention, demonstrated significantly improved retention-free survival in the GLP-1 RA combination group (log-rank *p* = 0.0064). The estimated hazard ratio for retention was 1.74 (95% CI: 1.16–2.59), indicating a significantly higher risk of urinary retention in the BTX-A-only group over the one-year follow-up period (Figure 1).

### 2.2. Urinary Tract Infection

For urinary tract infection (UTI) outcomes, 475 patients in the BTX-A-only cohort and 504 in the BTX-A + GLP-1 RA cohort were excluded due to a documented history of UTI prior to receiving BTX-A or BTX-A + GLP-1 RA in each group, respectively. Among the remaining patients, 69 of 517 (13.3%) in the BTX-A-only group developed a new UTI, compared to 43 of 488 (8.8%) in the GLP-1 combination group. This yielded an absolute risk difference of 4.54% (95% CI: 0.67–8.40%; *p* = 0.0224).

**Figure 2:**
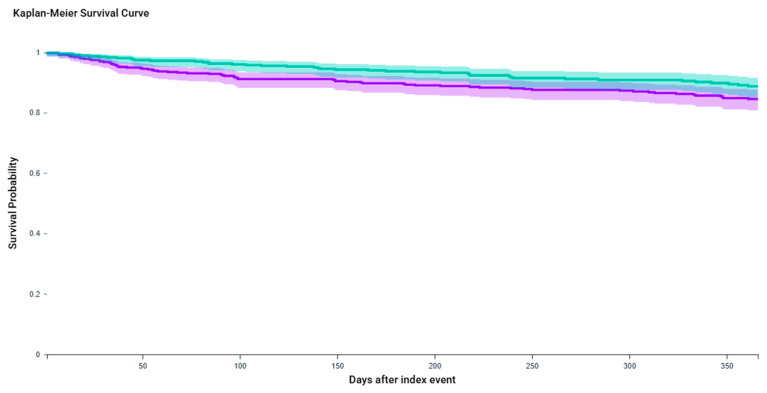
Kaplan–Meier curves for urinary tract infection (UTI)-free survival over one year following BTX-A injection, with or without concurrent GLP-1 receptor agonist therapy. The purple line represents the *OAB + BTX-A injection* cohort, while the teal line represents the *OAB + BTX-A + GLP-1 agonist/Semaglutide* cohort. Patients receiving combination therapy (OAB + BTX-A + GLP-1 RA) demonstrated significantly higher UTI-free survival compared to those receiving BTX-A alone (log-rank *p* = 0.042). At one year, survival probability was 88.7% in the combination group versus 84.5% in the BTX-A-only group. The hazard ratio for UTI in the BTX-A-only group was 1.48 (95% CI: 1.01–2.17), indicating a 48% increased risk relative to combination therapy.

KM analysis over one year demonstrated a higher UTI-free survival probability in the GLP-1 RA combination group (88.7%) compared to the BTX-A-only group (84.5%), with a statistically significant log-rank test (χ^2^ = 4.136, *p* = 0.0420) (Figure 2).

### 2.3. Urinary Antispasmodic Use

For urinary antispasmodic use, 848 patients in the BTX-A-only cohort and 875 in the GLP-1 combination cohort were excluded due to a history of prior antispasmodic use prior to receiving BTX-A or BTX-A + GLP-1 RA in each group, respectively. Among the remaining patients, 17 of 144 (11.8%) in the BTX-A-only antispasmodics post-treatment, compared to 12 of 117 (10.3%) in the GLP-1 group. This yielded an absolute risk difference of 1.55% (95% CI: −6.07% to 9.17%; *p* = 0.6921).

**Figure 3:**
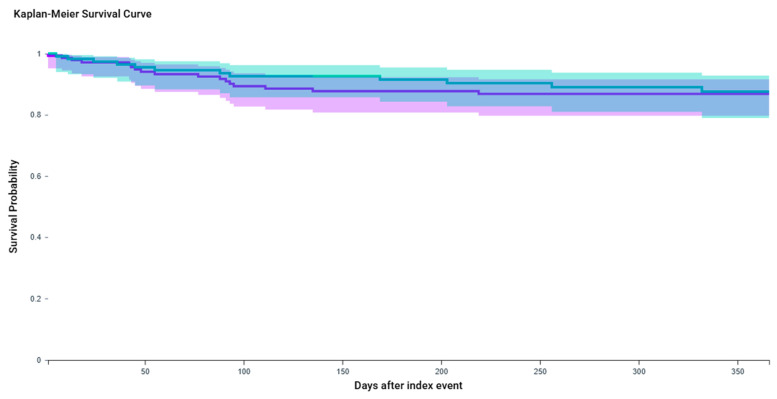
Kaplan–Meier curve for urinary antispasmodic-free survival among patients receiving BTX-A only versus BTX-A + GLP-1 receptor agonist therapy. The purple line represents the *OAB + BTX-A injection* cohort, while the teal line represents the *OAB + BTX-A + GLP-1 agonist/Semaglutide* cohort. After excluding patients with prior antispasmodic use (n = 848 in the BTX-A-only cohort and n = 875 in the combination cohort), 144 BTX-A-only patients and 117 BTX-A + GLP-1 patients remained for analysis. The 1-year antispasmodic-free survival probability was 86.85% in the BTX-A-only cohort and 87.59% in the combination cohort. No significant difference was observed between groups (log-rank *p* = 0.7234). The hazard ratio was 1.14 (95% CI: 0.55–2.39; *p* = 0.3984).

KM survival analysis demonstrated no significant difference in antispasmodic-free survival between cohorts over the three-year follow-up (log-rank *p* = 0.7234; HR 1.14, 95% CI: 0.55–2.39; *p* = 0.3984) (Figure 3).

## 3. Discussion

GLP-1 RA use, including semaglutide, was associated with reduced rates of urinary retention and UTIs in non-diabetic women receiving treatment for OAB, with nearly half the risk of retention (4.9% vs. 8.6%; *p* = 0.0044), a 4.5% absolute reduction in UTIs, and a better 1-year UTI-free survival (log-rank *p* = 0.0420) (Table 2). Survival analyses confirmed improved retention- and UTI-free outcomes in the GLP-1 cohort. These findings are consistent with prior literature suggesting that weight loss and systemic metabolic improvements can positively influence lower urinary tract function, including improving overactive bladder and urinary incontinence [30,31,32]. Additionally, our data adds to the ongoing debate over UTI risk and GLP-1 use [33,34,35]. No significant differences were observed in new antispasmodic use, suggesting benefits may be more specific to inflammatory or infectious outcomes rather than urgency symptoms. Overall, these findings highlight a potential protective role of GLP-1 RAs in bladder health independent of diabetes status.

To our knowledge, this study is the first to evaluate real-world urinary adverse events in a large non-diabetic OAB population treated with BTX-A and GLP-1 agonists. Recently, only one pilot survey had explored the impact of GLP-1 agonists on OAB symptoms [36]. Sandler et al. showed that respondents with more frequent baseline symptoms were more likely to report symptom improvement with semaglutide. Patients reported symptomatic improvement in OAB even among participants who did not experience significant weight loss during GLP-1 receptor agonist therapy, suggesting that the observed benefit may be independent of weight reduction [36]. This observation is biologically plausible, as GLP-1 receptor agonists exert several direct metabolic and anti-inflammatory effects beyond adiposity control. Mechanistically, GLP-1 activation improves endothelial and vascular function, reduces systemic and urothelial inflammation, modulates autonomic tone, and enhances natriuresis and diuresis through renal tubular actions. These early insights, although anecdotal and subject to recall bias, align with our findings.

Weight loss itself clearly plays a role in OAB symptom relief. Semaglutide trials show an average 11.85% weight loss vs. placebo, underscoring systemic therapeutic potential [37]. Given that obesity increases intra-abdominal pressure and weakens pelvic floor support, GLP-1-induced weight loss would alleviate the pathophysiologic contributors to OAB [38]. The American Urogynecology Society reviewed 43 publications (39 studies; 5 RCTs). They found high certainty evidence that behavioral weight loss yields modest urinary incontinence improvement at 1–2.9 years [30]. In addition to overweight populations, data from diabetic populations (versus simply overweight) show a positive correlation when measuring UTI risk, particularly in women [39,40].

In addition to OAB, GLP-1 receptor agonists have gained interest across a spectrum of urologic conditions including erectile dysfunction, prostate cancer, male fertility, and urolithiasis [41,42,43,44,45,46,47,48,49]. These studies, ranging from retrospective database analyses to prospective surveys and preclinical models, suggest mostly potential benefits with mechanisms often attributed to weight loss, central neural modulation, anti-inflammatory effects, or metabolic improvements.

Beyond weight loss, several studies have linked chronic inflammation to OAB. Elevated C-reactive protein levels have been observed in affected patients, correlating with worse OAB symptom scores [50,51,52]. It has been proposed that the loss of protective immunologic factors and increased cellular stress responses lead to heightened peripheral afferent nerve excitability and detrusor overactivity [53]. In support of this, preclinical studies in mouse models suggest that the antioxidant, resveratrol, can mitigate both systemic and bladder-specific oxidative stress, leading to improved bladder function [54]. UTIs, which are more prevalent in overweight individuals, may also contribute to this inflammatory cascade.

An intriguing, proposed mechanism of action for GLP-1 RAs, independent of their effects on obesity and inflammation, is neuromodulation. Studies suggest that, rather than acting primarily through GLP-1-producing neurons in the hindbrain or GLP-1 receptors in the vagus nerve, these drugs mediate weight loss via the arcuate nucleus (ARC) of the hypothalamus [55,56]. The ARC houses key regulators of appetite and metabolism, including pro-opiomelanocortin (POMC), neuropeptide Y (NPY), and agouti-related peptide (AgRP) neurons. Through its influence on the autonomic nervous system, the ARC modulates blood pressure, feeding behavior, glucose homeostasis, and innate immune responses [57]. Given this broad autonomic role, bladder control may also be affected by ARC dysfunction. Concurrently, metabolic syndrome and obesity, both known risk factors of OAB, may stem, in part, from ARC dysfunction. Notably, the extracellular perineuronal net surrounding ARC neurons undergoes remodeling in metabolic dysfunction [58]. Ultimately, if GLP-1 RAs can restore ARC neuronal function, they may offer therapeutic benefits even beyond metabolic regulation, potentially alleviating OAB symptoms.

One of the most common drawbacks to GLP-1 medication is the potential for gastrointestinal side effects [59]. This can be observed in our study with increased use of antiemetics (75.1% vs. 83.9%; *SMD* = 0.219; *p* < 0.0001) and laxatives (68.5% vs. 74.6%; *p* = 0.0028). Activation of GLP1 receptors in the area postrema (AP) of the brain drives nausea/aversion, while activation of the same receptors in the nucleus solitary tract plays a role in satiety [60].

Practically, these findings may help guide patient selection for BTX-A therapy. By identifying subgroups such as overweight or obese individuals using GLP-1 RAs who demonstrate more favorable risk-benefit profiles, physicians may be more inclined to recommend BTX-A treatment. This study also strengthens the viability of multimodal treatment in obese or overweight OAB patients, highlighting a tailored, more patient-centered approach to refractory disease. Ultimately, this work supports GLP-1 RA usage beyond metabolic indications in select urologic patients, such as those with treatment-resistant OAB, co-existing obesity, or heightened UTI risk.

On a larger scale, the public health implications of improving urinary symptoms are considerable. Urinary incontinence is associated with reduced mobility, impaired strength, and increased cardiovascular tone, all of which affect quality of life and independence, particularly in older women. Moreover, psychosocial burdens such as depression and social withdrawal further compound the clinical significance of OAB. Reducing incontinence-related events could therefore mitigate a spectrum of downstream complications.

This study has several notable strengths. It represents one of the largest real-world analyses to date evaluating BTX-A treatment outcomes in patients with and without GLP-1 RA use, enhancing its generalizability to routine clinical practice. The use of a large, geographically diverse dataset, TriNetX, allows for robust comparisons across patient subgroups and care settings. Rigorous adjustment for baseline covariates, including comorbidities and prior medication use, strengthens the internal validity of the findings. By focusing on a non-diabetic population, the study minimizes possible confounding effects of hyperglycemia on urinary adverse events. Furthermore, the inclusion of clinically meaningful outcomes such as urinary retention, UTI incidence, and antispasmodic use provides practical relevance for urologists and other clinicians managing OAB in a rapidly growing population of GLP-1 RA users.

Despite these promising findings, several barriers and limitations temper their clinical translation. GLP-1 receptor agonist therapies remain costly, resource-intensive, and often require specialist oversight. Growing demand, limited supply, and adverse effects such as gastrointestinal intolerance may further restrict their widespread adoption.

As a retrospective, real-world analysis, this study is subject to residual confounding despite propensity score adjustment. Key variables such as the degree of weight loss, medication adherence, timing and duration of GLP-1RA exposure relative to the BOTOX index event, and behavioral or lifestyle factors were unavailable and may have influenced the observed associations. Antimicrobial usage may have also affected urinary tract infection outcomes. Patient-reported outcomes, including urgency episodes and symptom severity, were also not captured, limiting assessment of subjective treatment response. Additionally, the dataset was not stratified by OAB subtype or symptom severity, which may affect the generalizability of these results. Detailed procedural data, including BOTOX dose, injection technique, and baseline bladder capacity, were inconsistently recorded and could impact both efficacy and adverse event rates.

Accordingly, these results should be interpreted as associative and hypothesis-generating rather than causal. Future research should prioritize prospective randomized controlled trials with extended follow-up to validate these observations and clarify the therapeutic role of GLP-1RAs in personalized OAB management. Such studies should incorporate validated symptom questionnaires and bladder diaries to capture patient-reported outcomes and assess clinical response more comprehensively. Further translational and basic science investigations are also warranted to elucidate whether GLP-1RAs influence bladder function via central ARC or peripheral mechanisms independent of metabolic effects.

Although our findings suggest a potential benefit of concurrent GLP-1 receptor agonist therapy in reducing urinary adverse events after BTX-A treatment, this combination is not yet clinically established or routinely applied in practice. Prior randomized trials, such as Amundsen et al., have shown that urinary tract infections and transient urinary retention are common following BTX-A, occurring in up to 35% and 8% of patients, respectively [61]. In this context, the comparatively lower incidence of these adverse events among patients receiving concomitant GLP-1RA therapy in our analysis may reflect a neuromodulatory influence of GLP-1 signaling on bladder or detrusor function; however, unmeasured confounders, including behavioral, metabolic, or pharmacologic factors, may also contribute to these associations. Future prospective randomized studies are needed to clarify its possible clinical utility.

## 4. Conclusions

In this large, real-world, propensity-matched analysis of non-diabetic patients with overactive bladder, concurrent use of GLP-1 receptor agonists was associated with a lower risk of urinary retention and UTI following BTX-A treatment.

Our findings are hypothesis-generating and suggest that GLP-1 receptor agonists may exert beneficial effects beyond weight loss. As the clinical use of GLP-1RAs continues to expand among overweight and obese populations, further prospective and mechanistic studies are warranted to validate these observations and to clarify the role of GLP-1RAs as a possible adjunctive strategy in the multimodal management of OAB.

## 5. Materials and Methods

We conducted a retrospective cohort study using the TriNetX Research Network, a federated health research platform that aggregates de-identified electronic health record (EHR) data from participating healthcare organizations worldwide. As of 7/05/2025, the total number of non-diabetic male patients on semaglutide or other GLP-1 RAs across 115/150 healthcare organizations (HCOs) was 184,014, while the number of non-diabetic female patients on semaglutide or other GLP-1 RAs across 118/150 HCOs was 504,514. Patients with overactive bladder (OAB) who received intravesical BTX-A injections were also identified. We then defined two cohorts for analysis: (1) OAB patients who received BTX-A alone and (2) OAB patients who received BTX-A in conjunction with a GLP-1 RA (semaglutide or equivalent).

Non-diabetic patients with OAB were initially identified using procedural and prescription data to capture BTX-A administration and GLP-1 RA exposure. Patients receiving both therapies within the same treatment period were assigned to the combination cohort. To minimize confounding, we performed 1:1 propensity score matching using a greedy nearest-neighbor algorithm with a caliper width of 0.1 pooled standard deviations. The covariates included in the propensity model were selected based on clinical relevance and potential influence on both treatment assignment and outcomes. These covariates comprised age at index (as a continuous variable), biological sex, race (White [2106-3], Black or African American [2054-5], Asian [2028-9]), and ethnicity (Hispanic or Latino [2135-2] and Not Hispanic or Latino [2186-5]). Additional variables included overweight and obesity status using ICD-10-CM codes E65–E68 and granular body mass index (BMI) categories defined by Z68.27 through Z68.42. We also adjusted for hypertensive comorbidity using ICD-10-CM codes I10–I11A. Covariate balance between treatment groups was assessed using standardized mean differences (*SMD*), with a threshold of <0.1 considered indicative of adequate balance. 

Primary outcomes included incidence of urinary tract infection (UTI), urinary retention, and urinary antispasmodic use following BTX-A treatment. These were defined using the following codes: UTI (ICD-10-CM N39.0; urinary retention (ICD-10-CM R33); antispasmodic use (VA drug class). The index event was defined by TriNetX as the date on which a patient first met *all* inclusion criteria for the cohort. For the BOTOX-only cohort, this corresponded to the first record of BTX-A injection (CPT 52287) with an overactive bladder diagnosis (ICD-10 N32.81). For the BTX-A + GLP-1RA cohort, the index event was the earliest date on which patients concurrently met the criteria for overactive bladder, BTX-A injection, and an active GLP-1 receptor agonist prescription (semaglutide or analogues). Thus, the index date represents the first encounter where both therapies overlapped in the electronic health record.

For each outcome, absolute risk, risk difference, risk ratio, and odds ratio were calculated with corresponding 95% confidence intervals. Time-to-event data were evaluated using one-year Kaplan–Meier (KM) survival analysis, with survival curves compared using log-rank tests with estimated Hazard ratios (HRs). Proportionality assumptions were verified for all survival models. Median follow-up times were also reported for each matched cohort to assess observation period comparability. Patients with pre-existing diagnoses of the respective outcome prior to the BTX-A/GLP-1 receptor agonist index date were excluded from each time-to-event analysis.

All analyses were performed within the TriNetX platform, which computes frequencies, summary statistics, covariate balance diagnostics, and outcome models using embedded statistical modules. This study was conducted in accordance with institutional and platform-level policies for research involving de-identified data and was deemed exempt from IRB review.

## Figures and Tables

**Table 1:** Baseline characteristics of patients receiving intradetrusor onabotulinumtoxinA (BOTOX) injection for overactive bladder (OAB), with and without concomitant glucagon-like peptide-1 (GLP-1) receptor agonist therapy (semaglutide). Values are shown before and after 1:1 propensity score matching. Continuous variables are presented as mean ± standard deviation (SD); categorical variables are presented as number (%). Body mass index (BMI) values reflect measurements at the index encounter: pre-BOTOX or pre-BTX-A + GLP-1 RA in each group, respectively.

Characteristic	OAB + BTX-A (Before Matching)	OAB + BTX-A + GLP1 RA (Before Matching)	*p*-Value (Before Matching)	OAB + BTX-A (After Matching)	OAB + BTX-A + GLP1 RA (After Matching)	*p*-Value (After Matching)
Age at Index (Mean ± SD)	62.4 ± 17.6	59.4 ± 13.2	<0.0001	59.3 ± 13.9	59.4 ± 13.2	0.8073
Female	15,971 (80.2%)	904 (91.1%)	<0.0001	913 (92.0%)	904 (91.1%)	0.4668
Male	3563 (17.9%)	63 (6.4%)	<0.0001	59 (5.9%)	63 (6.4%)	0.7085
Not Hispanic or Latino	15,513 (77.9%)	800 (80.6%)	0.0435	802 (80.8%)	800 (80.6%)	0.9093
White	15,858 (79.7%)	752 (75.8%)	0.0034	770 (77.6%)	752 (75.8%)	0.339
Black or African American	1706 (8.6%)	127 (12.8%)	<0.0001	115 (11.6%)	127 (12.8%)	0.4104
Hispanic or Latino	1333 (6.7%)	69 (7.0%)	0.7498	71 (7.2%)	69 (7.0%)	0.8608
Asian	339 (1.7%)	10 (1.0%)	0.0955	10 (1.0%)	10 (1.0%)	1
Overweight/Obesity	5216 (26.2%)	756 (76.2%)	<0.0001	755 (76.1%)	756 (76.2%)	0.958
Hypertension	8944 (44.9%)	575 (58.0%)	<0.0001	564 (56.9%)	575 (58.0%)	0.6175
Stress Incontinence	5398 (27.1%)	389 (39.2%)	<0.0001	391 (39.4%)	389 (39.2%)	0.9268
Body Mass Index (BMI) 40–44.9	880 (4.4%)	214 (21.6%)	<0.0001	219 (22.1%)	214 (21.6%)	0.7858
BMI 45–49.9	431 (2.2%)	110 (11.1%)	<0.0001	104 (10.5%)	110 (11.1%)	0.6641
BMI 29–29.9	453 (2.3%)	53 (5.3%)	<0.0001	48 (4.8%)	53 (5.3%)	0.6096
BMI 28–28.9	457 (2.3%)	40 (4.0%)	0.0005	37 (3.7%)	40 (4.0%)	0.7273
BMI 27–27.9	461 (2.3%)	37 (3.7%)	0.0044	38 (3.8%)	37 (3.7%)	0.9063
Antimicrobials	17,619 (88.5%)	976 (98.4%)	<0.0001	928 (93.5%)	976 (98.4%)	<0.0001
Antiemetics	12,021 (60.4%)	832 (83.9%)	<0.0001	745 (75.1%)	832 (83.9%)	<0.0001
Laxatives	11,381 (57.2%)	740 (74.6%)	<0.0001	680 (68.5%)	740 (74.6%)	0.0028
Anti-infectives, vaginal	7163 (36.0%)	585 (59.0%)	<0.0001	518 (52.2%)	585 (59.0%)	0.0025

**Table 2:** Summary of our study’s key findings.

Outcome	Event Rate (BTX-A Group)	Event Rate (BTX-A + GLP-1 Group)	Risk Difference (95% CI)	KM Log-Rank *p*	HR (95% CI)
Urinary Retention	8.60%	4.90%	3.66% (1.16–6.17%)	0.0064	1.74 (1.16–2.59)
UTI	13.30%	8.80%	4.54% (0.67–8.40%)	0.042	1.48 (1.01–2.17)
Antispasmodic Use	11.80%	10.30%	1.55% (−6.07 to 9.17%)	0.7234	1.14 (0.55–2.39)

## Data Availability

The original contributions presented in this study are included in the article. Further inquiries can be directed to the corresponding authors.

## Abbreviations

The following abbreviations are used in this manuscript:

OAB	Overactive bladder
UTI	Urinary Tract Infection
GLP-1 RA	Glucagon-Like Peptide-1 Receptor Agonist
BTX-A	OnabotulinumtoxinA
KM	Kaplan–Meier
BMI	Body Mass Index
ARC	Arcuate Nucleus
AP	Area Postrema
NTS	Nucleus Tractus Solitarius
POMC	Pro-Opiomelanocortin
NPY	Neuropeptide Y
AgRP	Agouti-Related Peptide
SMD	Standardized Mean Difference
HR	Hazard Ratio
FDA	U.S. Food and Drug Administration
PCOS	Polycystic Ovary Syndrome
AUA	American Urological Association
EAU	European Association of Urology
ICD-10-CM	International Classification of Diseases, 10th Revision, Clinical Modification
EHR	Electronic Health Record
IRB	Institutional Review Board
VA	Veterans Affairs (used in context of drug classes
IQR	Interquartile Range
CI	Confidence Interval
TriNetX	A federated real-world data research network/platform

## References

[1] Zhang L., Cai N., Mo L., Tian X., Liu H., Yu B. (2025). Global prevalence of overactive bladder: A systematic review and meta-analysis. Int. Urogynecol. J..

[2] UpToDate Urgency Urinary Incontinence/Overactive Bladder (OAB) in Females: Treatment. https://www.uptodate.com/contents/urgency-urinary-incontinence-overactive-bladder-oab-in-females-treatment.

[3] Feloney M.P., Stauss K., Leslie S.W. (2025). Sacral Neuromodulation. StatPearls.

[4] Orasanu B., Mahajan S.T. (2013). The use of botulinum toxin for the treatment of overactive bladder syndrome. Indian J. Urol..

[5] Cameron A.P., Chung D.E., Dielubanza E.J., Enemchukwu E., Ginsberg D.A., Helfand B.T., Linder B.J., Reynolds W.S., Rovner E.S., Souter L. (2024). The AUA/SUFU guideline on the diagnosis and treatment of idiopathic overactive bladder. J. Urol..

[6] Bonkat G., Kranz J., Cai T., Geerlings S.E., Köves B., Pilatz A., Medina-Polo J., Schneidewind L., Schubert S., Veeratterapillay R. EAU Guidelines. Proceedings of the EAU Annual Congress.

[7] Chen Y.H., Kuo J.H., Huang Y.T., Lai P.C., Ou Y.C., Lin Y.C. (2024). Evaluating the Efficacy and Safety of Botulinum Toxin in Treating Overactive Bladder in the Elderly: A Meta-Analysis with Trial Sequential Analysis of Randomized Controlled Trials. Toxins.

[8] Palm K.M., Abrams M.K., Sears S.B., Wherley S.D., Alfahmy A.M., Kamumbu S.A., Chakraborty N.N., Mahajan S.T., El-Nashar S.A., Henderson J.W. (2024). The Response of the Urinary Microbiome to Botox. Int. Urogynecol. J..

[9] Nitti V., Haag-Molkenteller C., Kennelly M., Chancellor M., Jenkins B., Schurch B. (2023). Treatment of neurogenic detrusor overactivity and overactive bladder with Botox (onabotulinumtoxinA): Development, insights, and impact. Medicine.

[10] Lo C.W., Wu M.Y., Yang S.S., Jaw F.S., Chang S.J. (2020). Comparing the Efficacy of OnabotulinumtoxinA, Sacral Neuromodulation, and Peripheral Tibial Nerve Stimulation as Third Line Treatment for the Management of Overactive Bladder Symptoms in Adults: Systematic Review and Network Meta-Analysis. Toxins.

[11] Hsieh P.F., Chiu H.C., Chen K.C., Chang C.H., Chou E.C.L. (2016). Botulinum toxin A for the treatment of overactive bladder. Toxins.

[12] Semins M.J., Shore A.D., Makary M.A., Weiner J., Matlaga B.R. (2012). The impact of obesity on urinary tract infection risk. Urology.

[13] Bausch K., Stangl F.P., Prieto J., Bonkat G., Kranz J. (2024). Urinary infection management in frail or comorbid older individuals. Eur. Urol. Focus..

[14] Rowe T.A., Juthani-Mehta M. (2013). Urinary tract infection in older adults. Aging Health.

[15] Hagovska M., Švihra J., Buková A., Horbacz A., Dračková D., Lupták J., Švihra J. (2020). The Relationship between Overweight and Overactive Bladder Symptoms. Obes. Facts..

[16] Khullar V., Sexton C.C., Thompson C.L., Milsom I., Bitoun C.E., Coyne K.S. (2014). The relationship between BMI and urinary incontinence subgroups: Results from EpiLUTS. Neurourol. Urodyn..

[17] Jing W., Wei C., Huang Y., Fu T., Shen W., Xiao W. (2025). Relationship between the weight-adjusted-waist index and urinary incontinence in women: A cross-sectional study of NHANES 2007 to 2020. Medicine.

[18] Zhang J., Chen W., Tang Z., Lin X., Wan X., Huang S., Luo H., Qian Y., He Z., Tang F. (2025). The Association between Obesity and Wet Overactive Bladder: Results from 2005 to 2020 National Health and Nutrition Examination Survey. Obes. Facts..

[19] Pomian A., Lisik W., Kosieradzki M., Barcz E. (2016). Obesity and Pelvic Floor Disorders: A Review of the Literature. Med. Sci. Monit..

[20] Tala M.R.Z., Ficky F., Lubis D.L., Mirsya Warli S. (2024). Efficacy of Body Weight Reduction in Improving Overactive Bladder Symptoms in Obese and Overweight Women: A Systematic Review. Nephro-Urol Mon..

[21] Collins L., Costello R.A. (2025). Glucagon-Like Peptide-1 Receptor Agonists. StatPearls.

[22] Xu D., Nair A., Sigston C., Ho C., Li J., Yang D., Liao X., Chen W., Kuang M., Li Y. (2022). Potential Roles of Glucagon-Like Peptide 1 Receptor Agonists (GLP-1 RAs) in Nondiabetic Populations. Cardiovasc Ther..

[23] Lincoff A.M., Brown-Frandsen K., Colhoun H.M., Deanfield J., Emerson S.S., Esbjerg S., Hardt-Lindberg S., Hovingh G.K., Kahn S.E., Kushner R.F. (2023). SELECT Trial Investigators. Semaglutide and cardiovascular outcomes in obesity without diabetes. N. Engl. J. Med..

[24] Heerspink H.J., Apperloo E., Davies M., Dicker D., Kandler K., Rosenstock J., Sørrig R., Lawson J., Zeuthen N., Cherney D. (2023). Effects of Semaglutide on Albuminuria and Kidney Function in People with Overweight or Obesity with or Without Type 2 Diabetes: Exploratory Analysis From the STEP 1, 2, and 3 Trials. Diabetes Care.

[25] Cena H., Chiovato L., Nappi R.E. (2020). Obesity, Polycystic Ovary Syndrome, and Infertility: A New Avenue for GLP-1 Receptor Agonists. J. Clin. Endocrinol. Metab..

[26] Jiang Z., Tan J., Yuan Y., Shen J., Chen Y. (2022). Semaglutide ameliorates lipopolysaccharide-induced acute lung injury through inhibiting HDAC5-mediated activation of NF-κB signaling pathway. Hum. Exp. Toxicol..

[27] Shnaien A., Mohammad A., Hassan E. (2023). Neuroprotective Effects of Semaglutide in Endotoxemia Mouse Model. Iran J. War Public Health.

[28] Tan S.A., Tan L. (2019). liraglutide and semaglutide attenuate inflammatory cytokines interferon-gamma, tumor necrosis factor-alpha, and interleukin-6: Possible mechanism of decreasing cardiovascular risk in diabetes mellitus. J. Am. Coll. Cardiol..

[29] Mosenzon O., Capehorn M.S., De Remigis A., Rasmussen S., Weimers P., Rosenstock J. (2022). Impact of semaglutide on high-sensitivity C-reactive protein: Exploratory patient-level analyses of SUSTAIN and PIONEER randomized clinical trials. Cardiovasc Diabetol..

[30] Yazdany T., Jakus-Waldman S., Jeppson P.C., Schimpf M.O., Yurteri-Kaplan L.A., Ferzandi T.R., Weber-LeBrun E., Knoepp L., Mamik M., Viswanathan M. (2020). American Urogynecologic Society Systematic Review: The Impact of Weight Loss Intervention on Lower Urinary Tract Symptoms and Urinary Incontinence in Overweight and Obese Women. Female Pelvic Med. Reconstr. Surg..

[31] Subak L.L., Wing R., West D.S., Franklin F., Vittinghoff E., Creasman J.M., Richter H.E., Myers D., Burgio K.L., Gorin A.A. (2009). Weight loss to treat urinary incontinence in overweight and obese women. N. Engl. J. Med..

[32] Zacche M.M., Giarenis I., Thiagamoorthy G., Robinson D., Cardozo L. (2017). Is there an association between aspects of the metabolic syndrome and overactive bladder? A prospective cohort study in women with lower urinary tract symptoms. Eur. J. Obstet. Gynecol. Reprod. Biol..

[33] Ljungberg C., Bredahl Kristensen F.P., Dalager-Pedersen M., Vandenbroucke-Grauls C., Sørensen H.T., Nørgaard M., Thomsen R.W. (2025). Risk of Urogenital Infections in People with Type 2 Diabetes Initiating SGLT2is Versus GLP-1RAs in Routine Clinical Care: A Danish Cohort Study. Diabetes Care.

[34] Dave C.V., Schneeweiss S., Kim D., Fralick M., Tong A., Patorno E. (2019). Sodium-Glucose Cotransporter-2 Inhibitors and the Risk for Severe Urinary Tract Infections: A Population-Based Cohort Study. Ann. Intern. Med..

[35] Soogoor A.R., Agrawal P., Pupo D., Kohn T.P., Du Comb W., Alshak M.N. (2024). MP45-11 Semaglutide Utilization in Weight Management and its Implications for Urinary Tract Infections and Urolithiasis. J. Urol..

[36] Sandler M.D., Williams A.D., Wein A., Amin K., Syan R. (2025). Effects of Glucagon like Peptide-1 agonists on patients with overactive bladder: A pilot study. Cont. Rep..

[37] Tan H.C., Dampil O.A., Marquez M.M. (2022). Efficacy and Safety of Semaglutide for Weight Loss in Obesity Without Diabetes: A Systematic Review and Meta-Analysis. J. ASEAN Fed. Endocr. Soc..

[38] Doumouchtsis S.K., Loganathan J., Pergialiotis V. (2022). The role of obesity on urinary incontinence and anal incontinence in women: A review. BJOG.

[39] Nitzan O., Elias M., Chazan B., Saliba W. (2015). Urinary tract infections in patients with type 2 diabetes mellitus: Review of prevalence, diagnosis, and management. Diabetes Metab. Syndr. Obes..

[40] Ahmed A.E., Abdelkarim S., Zenida M., Baiti M.A.H., Alhazmi A.A.Y., Alfaifi B.A.H., Majrabi R.Q.M., Khormi N.Q.M., Hakami A.A.A., Alqaari R.A.M. (2023). Prevalence and Associated Risk Factors of Urinary Tract Infection among Diabetic Patients: A Cross-Sectional Study. Healthcare.

[41] Able C., Liao B., Saffati G., Maremanda A., Applewhite J., Nasrallah A.A., Sonstein J., Alzweri L., Kohn T.P. (2025). Prescribing semaglutide for weight loss in non-diabetic, obese patients is associated with an increased risk of erectile dysfunction: A TriNetX database study. Int. J. Impot. Res..

[42] Varnum A.A., Pozzi E., Deebel N.A., Evans A., Eid N., Sadeghi-Nejad H., Ramasamy R. (2023). Impact of GLP-1 Agonists on Male Reproductive Health-A Narrative Review. Medicina.

[43] Alhajahjeh A., Al-Faouri R., Bahmad H.F., Bader T., Dobbs R.W., Abdulelah A.A., Abou-Kheir W., Davicioni E., Lee D.I., Shahait M. (2024). From Diabetes to Oncology: Glucagon-like Peptide-1 (GLP-1) Receptor Agonist’s Dual Role in Prostate Cancer. Cancers.

[44] Langroudi A.P., Chen A.L., Basran S., Sommer E.R., Stinson J., Cheng Y.-S., Del Giuduce F., Scott M., Eisenberg M.L. (2025). Male sexual dysfunction associated with GLP-1 receptor agonists: A cross-sectional analysis of FAERS data. Int. J. Impot. Res.

[45] Fang A., Frigo D.E., Hahn A., Razouki Z., Hwang J., Koutroumpakis E., Lawen T., Smith M., Hamilton-Reeves J., DiGiovanni J. (2025). GLP-1 Agonist Use Among Men with Localized Prostate Cancer: A Narrative Review and Rationale for Prospective Clinical Trials. Urology.

[46] Salvio G., Ciarloni A., Ambo N., Bordoni M., Perrone M., Rossi S., Balercia G. (2025). Effects of glucagon-like peptide 1 receptor agonists on testicular dysfunction: A systematic review and meta-analysis. Andrology.

[47] Nomiyama T., Kawanami T., Irie S., Hamaguchi Y., Terawaki Y., Murase K., Tsutsumi Y., Nagaishi R., Tanabe M., Morinaga H. (2014). Exendin-4, a GLP-1 receptor agonist, attenuates prostate cancer growth. Diabetes.

[48] Zhou L., Dong M., Feng G., Zhang Y., Wang J., Kang H., Dong Z., Ning J., Zhao Z., Wang C. (2024). Semaglutide mitigates testicular damage in diabetes by inhibiting ferroptosis. Biochem. Biophys. Res. Commun..

[49] Filippatos T.D., Panagiotopoulou T.V., Elisaf M.S. (2014). Adverse Effects of GLP-1 Receptor Agonists. Rev. Diabet. Stud..

[50] Chung S.D., Liu H.T., Lin H., Kuo H.C. (2011). Elevation of serum c-reactive protein in patients with OAB and IC/BPS implies chronic inflammation in the urinary bladder. Neurourol. Urodyn..

[51] Liu H.T., Jiang Y.H., Kuo H.C. (2013). Increased serum adipokines implicate chronic inflammation in the pathogenesis of overactive bladder syndrome refractory to antimuscarinic therapy. PLoS ONE.

[52] Pillalamarri N., Shalom D.F., Pilkinton M.L., Winkler H.A., Chatterjee P.K., Solanki M., Metz C.N. (2018). Inflammatory Urinary Cytokine Expression and Quality of Life in Patients with Overactive Bladder. Female Pelvic Med. Reconstr. Surg..

[53] Grundy L., Caldwell A., Brierley S.M. (2018). Mechanisms Underlying Overactive Bladder and Interstitial Cystitis/Painful Bladder Syndrome. Front. Neurosci..

[54] Meng X., Zhou J., Zhao C.N., Gan R.Y., Li H.B. (2020). Health Benefits and Molecular Mechanisms of Resveratrol: A Narrative Review. Foods.

[55] Singh I., Wang L., Xia B., Liu J., Tahiri A., El Ouaamari A., Wheeler M.B., Pang Z.P. (2022). Activation of arcuate nucleus glucagon-like peptide-1 receptor-expressing neurons suppresses food intake. Cell Biosci..

[56] Secher A., Jelsing J., Baquero A.F., Hecksher-Sørensen J., Cowley M.A., Dalbøge L.S., Hansen G., Grove K.L., Pyke C., Raun K. (2014). The arcuate nucleus mediates GLP-1 receptor agonist liraglutide-dependent weight loss. J. Clin. Investig..

[57] Jais A., Brüning J.C. (2022). Arcuate Nucleus-Dependent Regulation of Metabolism-Pathways to Obesity and Diabetes Mellitus. Endocr. Rev..

[58] Beddows C.A., Shi F., Horton A.L., Dalal S., Zhang P., Ling C.-C., Yong V.W., Loh K., Cho E., Karagiannis C. (2024). Pathogenic hypothalamic extracellular matrix promotes metabolic disease. Nature.

[59] Wright E.E., Aroda V.R. (2020). Clinical review of the efficacy and safety of oral semaglutide in patients with type 2 diabetes considered for injectable GLP-1 receptor agonist therapy or currently on insulin therapy. Postgrad Med..

[60] Huang K.-P., Acosta A.A., Ghidewon M.Y., McKnight A.D., Almeida M.S., Nyema N.T., Hanchak N.D., Patel N., Gbenou Y.S.K., Adriaenssens A.E. (2024). Dissociable hindbrain GLP1R circuits for satiety and aversion. Nature.

[61] Amundsen C.L., Richter H.E., Menefee S.A., Komesu Y.M., Arya L.A., Gregory W.T., Myers D.L., Zyczynski H.M., Vasavada S., Nolen T.L. (2016). OnabotulinumtoxinA vs. Sacral Neuromodulation on Refractory Urgency Urinary Incontinence in Women: A Randomized Clinical Trial. JAMA.

